# Transcription Factors in Prostate Cancer: Insights for Disease Development and Diagnostic and Therapeutic Approaches

**DOI:** 10.3390/genes15040450

**Published:** 2024-04-02

**Authors:** Karla C. S. Silva, Nadine Tambwe, Dalia H. Mahfouz, Martha Wium, Stefano Cacciatore, Juliano D. Paccez, Luiz F. Zerbini

**Affiliations:** 1 International Centre for Genetic Engineering and Biotechnology (ICGEB), Cape Town 7925, South Africa; karlasousa.silva@icgeb.org (K.C.S.S.); nadine.tambwe@icgeb.org (N.T.); dalia.hussein@icgeb.org (D.H.M.); mariet.wium@icgeb.org (M.W.); stefano.cacciatore@icgeb.org (S.C.); juliano.paccez@icgeb.org (J.D.P.); 2 Integrative Biomedical Sciences Division, Faculty of Health Sciences, University of Cape Town, Cape Town 7925, South Africa

**Keywords:** transcription factors, prostate cancer, diagnosis, prognosis, therapy

## Abstract

Transcription factors (TFs) are proteins essential for the regulation of gene expression, and they regulate the genes involved in different cellular processes, such as proliferation, differentiation, survival, and apoptosis. Although their expression is essential in normal physiological conditions, abnormal regulation of TFs plays critical role in several diseases, including cancer. In prostate cancer, the most common malignancy in men, TFs are known to play crucial roles in the initiation, progression, and resistance to therapy of the disease. Understanding the interplay between these TFs and their downstream targets provides insights into the molecular basis of prostate cancer pathogenesis. In this review, we discuss the involvement of key TFs, including the E26 Transformation-Specific (ETS) Family (ERG and SPDEF), NF-κB, Activating Protein-1 (AP-1), MYC, and androgen receptor (AR), in prostate cancer while focusing on the molecular mechanisms involved in prostate cancer development. We also discuss emerging diagnostic strategies, early detection, and risk stratification using TFs. Furthermore, we explore the development of therapeutic interventions targeting TF pathways, including the use of small molecule inhibitors, gene therapies, and immunotherapies, aimed at disrupting oncogenic TF signaling and improving patient outcomes. Understanding the complex regulation of TFs in prostate cancer provides valuable insights into disease biology, which ultimately may lead to advancing precision approaches for patients.

## 1. Introduction

According to the last Global Cancer Observatory (GLOBOCAN) report, approximately 20 million people are affected by some type of cancer manifestation. Prostate cancer (PCa) is the second most common type of cancer and holds the second highest rate of mortality among men worldwide [1]. Researchers globally commit their efforts to discovering effective treatments and more accurate diagnostic tools to address the challenges posed by PCa. Achieving this goal requires a comprehensive understanding of the mechanistic aspects of PCa. Regulation of protein expression is a complex event that involves tight regulation of different components. One class of proteins essential in protein expression is transcription factors (TFs). TFs play a critical role in the regulation of genes during homeostasis and across various cancer types. TFs recognize and bind directly to specific DNA sequences, resulting in direct and/or indirect activation of downstream genes [2,3]. 

PCa exhibits remarkable diversity in transcription factor involvement, reflecting the complexity of its pathogenesis and heterogeneity across patients. Transcription factors play pivotal roles in modulating gene expression programs that drive various aspects of PCa development, including proliferation, survival, differentiation, and metastasis. Key transcription factors implicated in PCa encompass a diverse array of families. The role of TFs in PCa is highly diverse. For instance, pioneer factors, such as FOXA1, are also targeted for therapeutic purposes in PCa [4]. In addition, TP53, RB1, and BRCA1-2 play an important role as tumor suppressors. Interestingly, these genes are commonly mutated in cancer patients, and the loss of their function often results in a more aggressive cancer phenotype. Although not acting mechanistically as a TF, BRCA1-2 directly influence TFs, such as AR [5] and MYC [6]. Given their importance, the discussion surrounding their roles in cancer and their potential as therapy targets is a topic of significant debate [7,8,9,10,11,12,13].

In this review, we will discuss the roles of TFs that play direct and significant roles in regulating key cellular processes in PCa, thus providing information regarding the molecular interactions that drive disease progression. We provide a significant discussion on SPDEF (SAM Pointed Domain Containing ETS Transcription Factor), ERG, androgen receptor (AR), Activating Protein-1 (AP-1), NF-κB (nuclear factor kappa-light-chain-enhancer of activated B cells), and MYC in PCa development, focusing on the molecular mechanisms involved in PCa development as well as their use in diagnostics and therapeutic approaches.

## 2. The ETS Family

**Figure 1:**
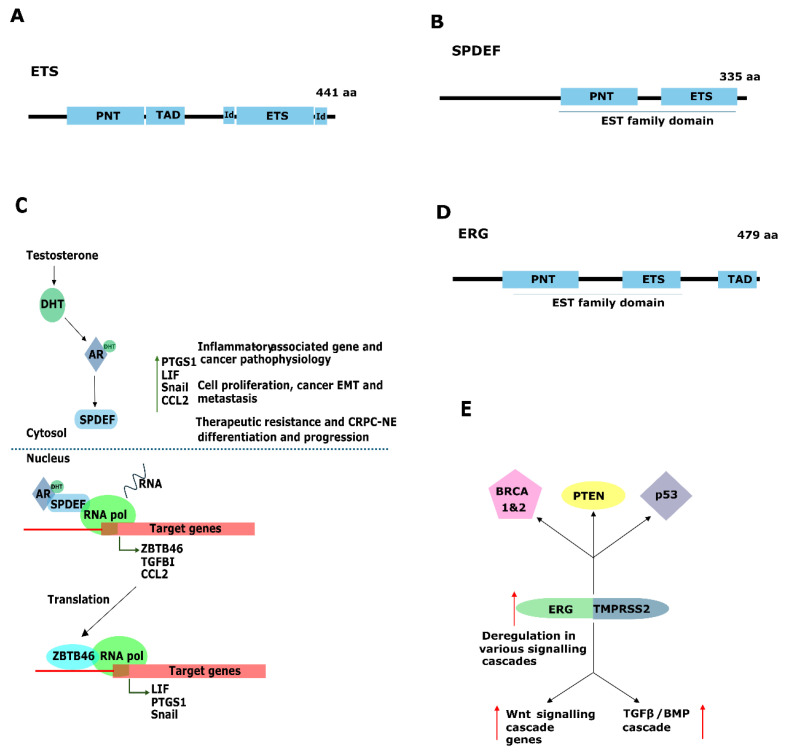
Representation of the ETS family of transcription factor SPDEF and ERG. (**A**) General structure of the ETS family of TFs showing the PNT (SAM pointed domain, which mediates protein–protein interactions within the ETS family) and the ETS (E26 transformation-specific) flanked by the Id region (interaction domains). (**B**) The SPDEF protein structure, including the PNT domain responsible for mediating protein–protein interactions within the ETS family, in addition to an ETS domain. (**C**) Example crosstalk between AR and SPDEF. Interaction of DHT and AR leads to activation of SPDEF and its transport to the nucleus. The complex AR/SPDEF interacts with RNApol, activating the transcription of target genes, such as ZBTB46, TGFBI, and CCL2. In turn, SPDEF targets genes, such as ZBTB46, leading to upregulation of PTGS1, LIF, Snail, and CCL2 proteins involved in processes, such as tumor progression and metastasis. SPEDF activation leads to activation of inflammatory-associated gene and cancer pathophysiology, cell proliferation, EMT, and metastasis that result in CRPC-NE differentiation and progression. (**D**) The ERG protein structure features a PNT domain, an ETS domain, and a TAD domain (transactivation domain). (**E**) TMPRSS2-ERG fusion. TMPRSS2-ERG fusion occurs in both normal and tumor cells. In prostate cancer, genomic rearrangement leads to TMPRSS2-ERG fusion, resulting in overexpression of genes associated with WNT and TGFB1/BMP transduction cascades and deregulation of tumor suppressor genes, such as BRCA1, BRCA2, p53, and PTEN. (Abbreviations: CRPC-NE, castration-resistant prostate cancer with neuroendocrine differentiation; EMT, Epithelial-to-Mesenchymal Transition).

The oncogenes family, ETS, is composed of 28 members divided into 11 sub-families that function as TFs in cells [14]. They share a conserved DNA-binding domain (DBD) with a helix–turn–helix structure named the ETS domain (Figure 1A) [14]. The transactivation of ETS factors is regulated by phosphorylation as well as interactions with other TFs [15]. They have key roles in essential physiological processes, such as hematopoiesis, angiogenesis, organogenesis, tissue development, differentiation, cell-cycle control, and cell proliferation, acting as transcriptional enhancers and repressors [4]. Interestingly, members of the ETS family are linked to oncogenesis because deregulation causes cellular transformation. Furthermore, differential expression or activation of various members of the ETS family is known to play a role in tumor progression and metastasis of cancer [15].

### 2.1. SPDEF in PCa, the Double Agent

SPDEF (SAM Pointed Domain Containing ETS Transcription Factor), also called prostate-derived ETS factor (PDEF), has a vital function not only in normal cell development but also in cancer development. SPDEF is a member of the ETS transcription factor family, which controls important cellular processes, such as cytoskeleton organization and cell cycle progression [16,17]. Like all members of the ETS, SPDEF contains the highly conserved ETS DNA binding domain; however, the N-terminal SAM or pointed (PNT) domain that aids protein–protein interactions and dimerization in SPDEF shows weak homology. Therefore, SPDEF only binds to the GGAT DNA motif rather than the GGAA DNA motif (Figure 1B) [18]. SPDEF expression in tumor tissue is organ- as well as subtype-specific. In lung, brain, metastatic gastric, and ovarian cancer, SPDEF expression is higher compared to the normal organ tissue [19,20,21] and acts as an oncogene, promoting proliferation, migration, and invasion in other cancer types, like head-and-neck squamous cancer [22], and in hepatic carcinoma decreased expression is reported [17,23].

SPDEF has a dual role in cancer by potentializing or inhibiting and acting upon features, such as adhesion, migration, invasion, and epithelial–mesenchymal transition (EMT) [16,17]. The loss of function of SPDEF combined with TGFBI and androgen-deprivation therapy (ADT) promotes EMT and bone metastasis [24]. In contrast, the overexpression of SPDEF leads to a reduction in mRNA and protein levels of TF Foxm1, leading to tumor proliferation. Targeting Foxm1 through SPDEF may be an option to disrupt the positive feedback loop that accelerates tumor cell proliferation (Figure 1C) [25].

The chemokine C–C motif ligand 2 (CCL2) is essential in recruiting tumor-associated macrophages. It mediates EMT induced by ADT and reduces the transcriptional repressor SPDEF. In tissues from PCa patients receiving ADT, low SPDEF levels were correlated with high CCL2 expression compared to treatment-naïve patients [26,27].

The interplay between SPDEF and other TFs, signaling pathways, and microenvironmental cues likely determines the effect of SPDEF on cancer cell behavior. In PCa, SPDEF was first reported as an oncogenic driver [18,28]. However, in other reports, SPDEF has been identified as a tumor suppressor, especially in metastatic PCa cases [25,29,30,31].

SPDEF is significantly upregulated in PCa tissue compared to normal epithelial tissue and is associated with Gleason grade, lymph node metastasis, prostate-specific antigen (PSA), and aggressive behavior [32]. Also, SPDEF is more frequently overexpressed in ERG (ETS-related Gene)-positive PCa than in ERG-negative cases [32]. Interestingly, the androgen regulates NK3 Homeobox 1 (NKX3.1) gene, whose expression is predominantly localized in the prostate epithelium and has a close correlation with SPDEF function in PCa. It acts as a negative regulator of cell growth in prostate tissue. NKX3.1 was identified as a SPDEF partner that inactivates SPDEF transcriptional activation [33]. Moreover, our group demonstrated that in PCa cell lines, SPDEF inhibition results in morphological cell changes accompanied by decreased epithelial markers, increased mesenchymal markers, decreasing cell adhesion, and increasing invasion and migration through activation of the TGFβ pathway [29]. In addition, we demonstrated that SPDEF protein stability and its effects on progression and metastasis are regulated by the CDK11p58/GADD45α/γ pathway [34]. 

#### Applications of SPDEF in Prostate Cancer Detection

SPDEF plays a crucial role in regulating genes associated with PCa progression and metastasis, making it a potential biomarker for risk stratification and treatment decisions. For instance, the detection of TMPRSS2 (Transmembrane Protease Serine 2) linked to SPDEF in urine can be an indicator of the aggressiveness and metastatic potential of PCa tumors [35,36]. 

RNA expression of SPDEF, ERG, and Prostate Cancer Antigen 3 (PCA3) detected in urine samples outperformed mainstream markers in the prediction of PCa with high sensitivity and specificity to PCa [37]. The RNA levels of these same genes (*SPDEF*, *ERG*, and *PCA3*) when isolated from urine-derived exosomes can be used to predict the probability of a high-grade PCa without the necessity of a digital rectal exam (DRE) [36,38,39,40,41]. The evaluation of these markers allied with the standard clinical criteria analysis increased by 30% the detection of high-grade PCa, thus improving patient stratification and medical practitioners’ decision making [42].

### 2.2. ERG and ERG Translocations

The *ERG* gene is located on chromosome 21q22 (Figure 1D) and is expressed in mesodermal cells, hematopoietic stem cells, and endothelial cells. ERG overexpression is known to inhibit programmed cell death and induce cell transformation and tumor development [43]. Once activated, ERG can modulate several signaling pathways implicated in PCa progression. It has been shown to upregulate the expression of Phosphoinositide 3-Kinase (PI3K) pathway components, leading to increased activation of downstream signaling molecules and enhanced tumor growth. The *ERG* gene plays a role in PCa development and is upregulated in metastatic PCa [44,45]. In PCa, ERG activates proteins, such as WNT, EZH2, TGFB, and SOX9 [46,47], and is known to induce NF-κB-mediated transcription through phosphorylation of NF-κB p65 [48], thus playing an important role in inducing the invasion of PCa cells. Moreover, ERG can affect EMT, a process implicated in cancer metastasis. ERG expression has been associated with the induction of EMT-related genes, such as FZD4, and loss of epithelial markers, such as E-cadherin [46,49,50]. Also, transactivation of vimentin (another EMT marker) mediated by ERG is known to increase cell mobility and invasion, thus promoting a more aggressive phenotype in PCa cells.

However, the most well-known mechanism of ERG activation in PCa is through gene fusions. In fact, gene rearrangement is observed in the majority (57–80%) of PCa tissue samples, and the TMPRSS2/ERG rearrangement in particular is present in 50% of prostate tumors [44,45]. TMPRSS2 is a type II transmembrane serine protease expressed in epithelial cells associated with physiological and pathological processes. Interestingly, TMPRSS2 is expressed at higher levels in the prostate when compared to other tissues [51]. The androgen-responsive promoter elements of TMPRSS2 mediate the overexpression of ETS family members in PCa. The TMPRSS2/ERG fusion results in the overexpression of ERG through the fusion of the 5′ untranslated region of the androgen-regulated *TMPRSS2* gene to the *ERG* gene, resulting in a full-length ERG protein altering the expression pattern and function of ERG [44,45] (Figure 1E). Mechanistically, TMPRSS2/ERG has been demonstrated to play a causal role in the transformation of the prostate epithelium as it activates cell invasion programs that displace basal cells through neoplastic transformation of the prostate epithelium [52]. Also, TMPRSS2-ERG fusion together with the loss of the tumor suppressor Phosphatase and Tensin homolog (PTEN) cooperates with the initiation of neoplasia [53]. Loss of PTEN causes overactivation of Akt, which is viewed as an indirect physiological target of PTEN as regulation of PI3K is linked to increased tumor angiogenesis, reduced apoptosis, and uncontrolled cell proliferation [54,55,56]. In this context, the PI3K/AKT pathway is activated and cooperates with TMPRSS2-ERG fusion to regulate the expression of genes involved in cancer initiation of neoplasia and proliferation as well as in EMT. Additionally, ERG fusion is known to synergize with K-Ras to promote invasion and suppress senescence in a TP53-independent manner [57]. Also, ERG interacts with the AR and enhances its transcriptional activity, thus promoting the expression of genes involved in cell proliferation and survival [58,59]. Finally, evidence obtained in PCa cell lines demonstrated [48] that co-binding of ERG and AP-1 elicits a synergistic effect on transcriptional activation [45].

#### Targeting ERG and ERG Translocations for Diagnostic and Therapeutic Purposes

As previously discussed, TMPRSS2/ERG rearrangement is present in about half of prostate tumors [53] and is associated with distinct clinicopathological features, including younger age at diagnosis, higher Gleason score, and more aggressive disease behavior, highlighting its potential prognostic significance as ERG fusions are prevalent in both primary and advanced PCa. In this sense, the detection of ERG rearrangements has emerged as a promising diagnostic marker in PCa [60,61,62,63,64]. Techniques, such as fluorescence in situ hybridization (FISH), immunohistochemistry (IHC), and reverse transcription-polymerase chain reaction (RT-PCR), are employed for detecting ERG fusion events in clinical specimens [65,66]. However, the prognostic value of ERG expression in PCa is still not fully understood and may depend on several factors, including patient heterogeneity and the methods used for detection. Studies evaluating ERG as a prognostic biomarker have shown more consistent results in cohorts assessing disease progression from precursor lesions or during active surveillance. High-grade prostatic intraepithelial neoplasia (PIN) patients with ERG overexpression have a higher risk of PCa progression compared to ERG-negative patients, indicating that ERG positivity may correlate with increased disease progression and a higher incidence of PCa-specific death [67]. Furthermore, recently, it was found that the detection of TMPRSS2-ERG fusion in circulating tumor cells of castration-resistant prostate cancer (CRPC) patients may predict resistance to taxanes [68,69].

Nonetheless, no clear association has been observed between ERG fusions and other genetic alterations implicated in PCa initiation and progression. However, clinical trials are in the course of evaluating ERG fusions in the prognostic and predictive values at different stages of the disease as well as during treatment (NCT02573636) (see Table 1). TMPRSS2-ERG fusion serves as a cancer-specific biomarker for early PCa diagnosis and can be detected non-invasively in urine samples, thus potentially reducing overtreatment. Initial attempts to target ERG involved PARP inhibitors, showing promise in preclinical models but failing in clinical trials for metastatic castration-resistant prostate cancer (mCRPC) patients. HDAC inhibitors also showed promise in preclinical models but failed in clinical trials. Additionally, small molecule inhibitors targeting ERG directly have been developed, such as YK-4-279, DB1255, VPC-18005, and ERGi-USU, with varying success in preclinical models [69,70,71,72,73]. Alternative approaches include RNA interference and peptides targeting ERG, showing efficacy in reducing tumor growth in preclinical models. However, despite these efforts, there is still a lack of a specific therapeutic strategy targeting ERG in clinical practice (Figure 1E, Table 1).

Additionally, therapeutic strategies targeting ERG-positive PCa are currently under investigation. The identification of synthetic lethal interactions through advanced genomic and epigenomic characterization could reveal novel therapeutic targets. CRISPR-based technologies, utilizing intronic genomic breakpoints from gene rearrangements, present a genotype-specific approach for personalized treatment. Computational approaches and proteolysis-targeting chimeras (PROTACs) offer additional strategies [74,75]. These diverse approaches hold promise for future therapies targeting ERG-positive PCa.

## 3. Androgen Receptor, the Great Player

**Figure 2:**
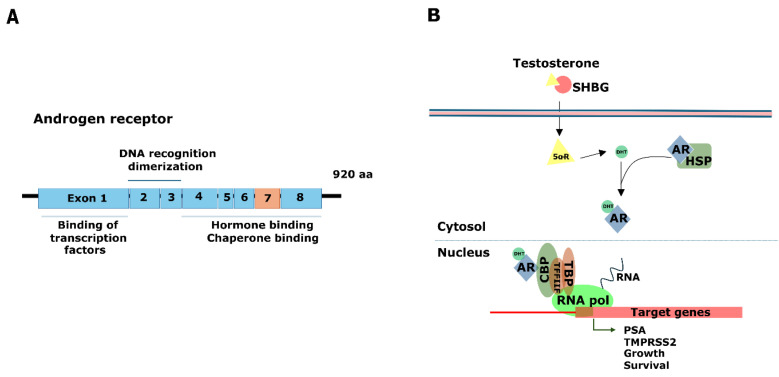
Androgen receptor structure and signaling pathway in PCa. (**A**) Representation of protein domains of AR. It comprises domains for transcription factor binding, DNA recognition, hormone binding/chaperone binding, and a nuclear localization signal (NLS). (**B**) Example crosstalk between AR and SPDEF. Interaction of DHT and AR leads to activation of SPDEF and its transport to the nucleus. The complex AR/SPDEF interacts with RNApol, thus activating the transcription of target genes, such as ZBTB46, TGFBI, and CCL2. In turn, SPDEF targets genes, such as *ZBTB46*, leading to upregulation of PTGS1, LIF, Snail, and CCL2 proteins involved in certain processes, such as tumor progression and metastasis. SPEDF activation leads to the activation of inflammatory-associated gene and cancer pathophysiology, cell proliferation, EMT, and metastasis that result in CRPC-NE differentiation and progression.

The AR plays a significant role in regulating growth, differentiation, angiogenesis, and metabolism in both normal prostate and human PCa. It is classified as a nuclear steroid receptor and is transcribed from the *AR* gene situated on Chromosome Xq11-12 [76]. It is associated with other receptors, such as estrogen receptor (ER), glucocorticoid receptor (GR), progesterone receptor, and mineralocorticoid receptor [77,78,79]. Binding of AR to its natural ligands, testosterone and 5α-dihydrotestosterone (DHT), starts the development and differentiation of male sexuality. AR has been shown to play a significant role in biological activities in the bone, muscle, prostate, and adipose tissue and the reproductive, cardiovascular, immunological, neurological, and hemopoietic systems [77,80]. Three primary functional domains are thought to make up the structure of AR: the most variable N-terminal transcriptional regulatory domain, the highly conserved DNA binding domain (DBD), and, lastly, the ligand binding domain (Figure 2A) [81,82].

AR is the main signaling pathway involved in both the development of PCa and normal prostatic growth. It plays a critical role in PCa by regulating the development and normal physiology of the prostate gland, it mediates the pathology associated with cancer development and progression, and it determines the progression to advanced metastatic PCa by driving tumor survival (Figure 2B) [83]. Activation of AR requires binding to specific ligands, receptor dimerization, phosphorylation, and translocation into the nucleus, where it interacts with its coregulatory proteins to recognize response elements located in the proximal or distal intragenic and intergenic regions of androgen target genes, such as KLK3, NKX3.1, FKBP5, and TMPRSS2-ERG, which promotes prostate development and cancer progression [84,85]. 

### 3.1. AR’s Diagnostic and Therapeutic Applications

ADT is the first-line systemic therapeutic approach for patients with high-risk localized, recurrent, or advanced PCa. It can be administered through chemical methods or surgical castration and works by blocking the ARs from interacting with AR signaling molecules, resulting in apoptosis of malignant cells and inhibition of disease progression [76,84,86]. However, AR-targeting ADT treatment commonly leads to the development of resistant cells.

Castration resistance arises from a series of complex molecular events, including oncogene activation, inactivation of tumor suppressor genes, evasion of apoptosis, intra-tumoral androgen production, and abnormal AR activation [76]. In CRPC, AR is believed to be constitutively active, hypersensitive to androgens, or activated through non-canonical pathways, which leads to AR activation even in the presence of low levels of androgens. In addition, tumor cells in CRPC may exhibit increased extragonadal androgen production and AR activation independent of ligands.

### 3.2. Mechanism of Resistance Mediated by AR

In patients with CRPC, a primary therapeutic intervention is to directly target the AR. The AR pathway inhibitors, such as Abiraterone, which inhibit androgen synthesis, and Enzalutamide, which prevents AR activation, have emerged as foundational treatments in PCa treatment. As a result, these medications are effective in managing CRPC because they disrupt AR signaling, thus preventing the nuclear translocation of AR, and while they do not exhibit agonistic properties, resistance to this class of compounds has already emerged [83,87]. The resistance to Abiraterone and anti-androgens is mediated by mechanisms, such as AR overexpression, AR splice variants that enable ligand-independent AR transactivation, and changes in the expression and recruitment of AR coregulators (Figure 2B) [70]. 

The AR splice variant 7 (AR-V7) lacks the ligand-binding domain, which is a necessary component for constitutive activity and has an exon 7 deletion. As seen in Figure 2A, the presence of AR-V7 has been linked to a lower overall survival rates in patients receiving Enzalutamide or Abiraterone treatment, and it is implicated in the development of CRPC [88]. Interestingly, AR-V7 may be a predictive biomarker for therapy selection in men with metastatic mCRPC because patients positive for AR-V7 subjected to taxane therapy present better survival outcomes [89,90,91]. Additionally, AR splice variants lacking LBD may have therapeutic significance because they do not require androgens for activation [79]. Furthermore, AR variants can exhibit ligand-independent constitutive activity. The signaling of AR may also work together with other oncogenic pathways associated with EMT and cell survival to promote progression to metastatic CRPC.

Novel approaches to eradicate AR signaling are constantly being explored. Strategies target the inhibition of receptor-ligand binding or blocking the expression of proteins downstream to the AR signaling pathway. Another strategy employed is the use of PROTACs, small molecules that selectively degrade target proteins through the ubiquitin-proteasome system [92]. In fact, clinical trials using PROTACs like ARV-110 (NCT03888612) and ARV-766 (NCT05067140) are ongoing (Table 1), and results may represent the development of novel therapeutic agents for mCRPC. 

An additional treatment option for AR is to suppress AR gene expression at the transcriptional level. In this context, Wang and collaborators demonstrated that the nuclear receptor named RAR-related orphan receptor γ (RORγ) regulated AR expression in mCRPC through transcriptional inhibition [93]. Additionally, they demonstrated that RORγ antagonists XY018 and SR2211 reduce the growth of PCa tumors resistant to Enzalutamide, suggesting that this strategy may be able to get around second-generation antiandrogen resistance [87]. Nevertheless, no comprehensive analysis of AR binding between primary and resistant tumor specimens has been conducted yet. 

### 3.3. Neuroendocrine Prostate Cancer (NEPC) Transcription Factors

NEPC, categorized as an aggressive variant of prostate cancer (AVPC), typically emerges in advanced stages of the disease as a response to ADT, displaying neuroendocrine features while reducing adenocarcinoma luminal traits. NEPC tumors are less dependent on the canonical AR signaling, highly spread, and may progress with low or any rise in PSA levels [94,95]. Under the genomic perspective, it has been reported that in general, NEPC shows *TMPRSS2-ERG* gene fusion [96] and loss of tumor suppressors TP53 and RB1 [97]. Due to its particularities, NEPC also presents diverse set of TFs as players in its development and pathology. 

The aberrant expression of the TF ONECUT2 contributes to the development of neuroendocrine features in CRPC by downregulating AR and FOXA1 signaling [98]. Targeting ONECUT2 for therapeutic ends may lead to unfavorable side effects; on the other hand, ONECUT2-dependent tumor hypoxia can be more effective for NEPC patients [99]. 

Achaete-scute homolog-1 (ASCL1) is a pro-neural TF that has recently been found to trigger changes in chromatin through epigenetic remodeling in NEPC, promoting the activity of neuronal stem cells by modulating the enhancer of zeste homolog 2 (EZH2) [100], the catalytic subunit of polycomb repressive complex 2 (PRC2). EZH2 has already been considered a target for cancer therapy, as reviewed by Duan and colleagues [101], and recent efforts suggest that ASCL1 can also be a valuable clinical target in NEPC [100].

SOX2 (Sry-related HMG box-2) is mostly known for its role in reprogramming somatic cells into induced pluripotent stem cells (iPSCs) [102]. Its expression starts during early embryo development and increases considerably between the morula and blastocyst stages [103]. In NEPC, SOX2 has the potential to decrease the expression of specific adenocarcinoma prostate cancer genes while slightly contributing to the expression of neuroendocrine markers in vitro [104]. Also, SOX2 can promote tumorigenesis, metastasis, and drug resistance [105]. SOX2 is considered undruggable; however, several approaches have been administered to target SOX2 for cancer therapeutics, as reviewed by Zhang and colleagues [105].

NEUROD1 is a neuronal TF able to convert epithelial cells into neurons [106]. Alongside ASCL1, it significantly contributes to the promotion of malignant behavior and survival of human small cell lung carcinoma (SCLC) cell lines [107]. Therapy-induced NEPC can be categorized into subtypes based on the expression profiles of ASCL1 and NEUROD1 [108].

## 4. Activating Protein-1

**Figure 3:**
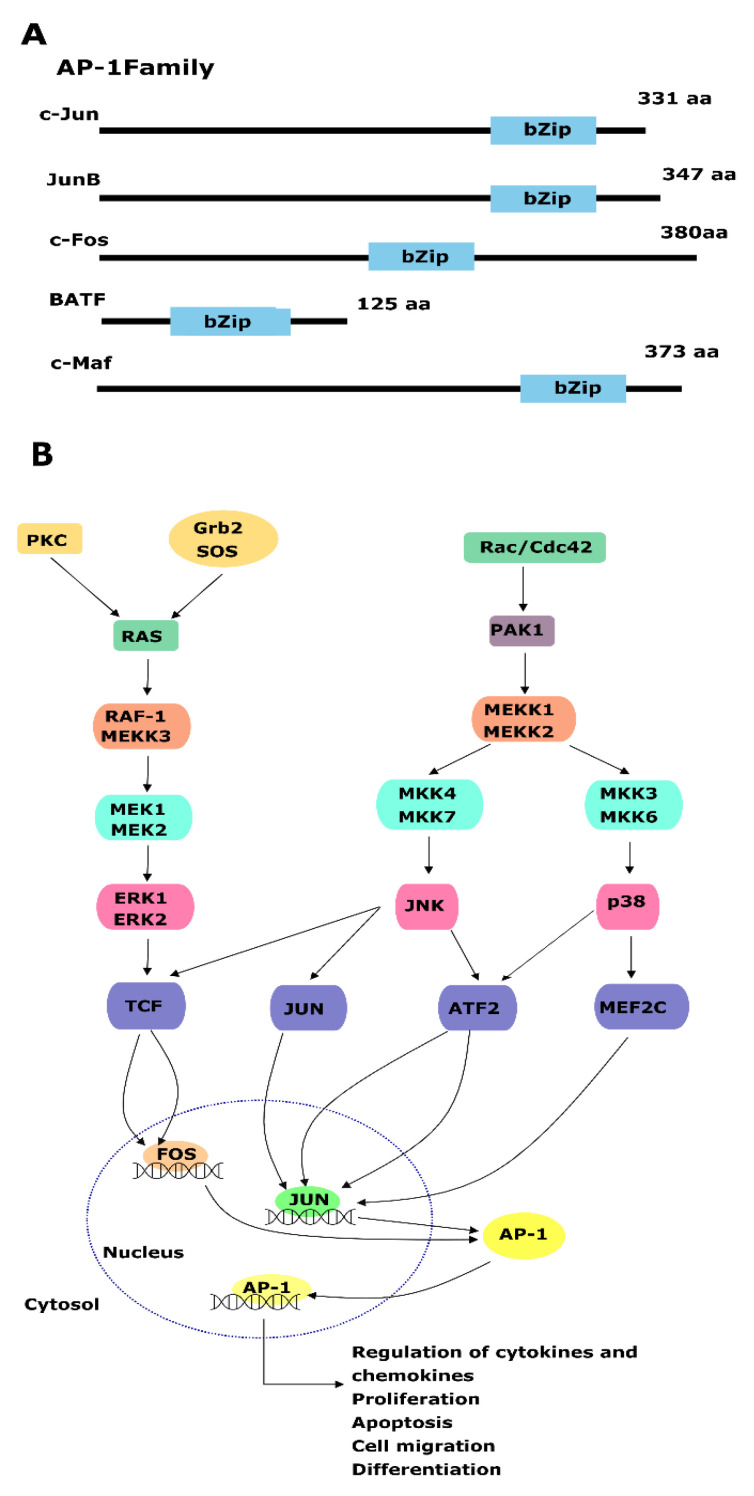
Representation of the diversity of the AP-1 family of TF. (**A**) Members of the AP-1 family, including C-Jun, JunB, c-Fos, BATF, and C-Maf, share a conserved bZip (basic leucine zipper) domain and its activation. (**B**) External stimuli, including growth factors, TNFα, and cytokines, activate G proteins, initiating MEK1/MEK2, MKK3/MKK6, and MKK3/MKK6 cascades. These cascades culminate in the transcriptional activation of AP-1, a transcription factor that regulates various cellular processes, such as growth, cell migration, differentiation, and proliferation pathways.

AP-1 is a family of TFs including four sub-families of hetero- and homo-dimers (Figure 3A), the Jun (c-Jun, JunB, JunD), Fos (c-Fos, FosB, Fra1, Fra2), BATF (BATF, BATF2, BATF3), and Maf (c-Maf, MafB, MafA) [109]. AP-1 TFs regulate a variety of cellular processes, such as cell division, apoptosis, senescence, differentiation, cell migration, immunity, oncogenic processes, and inflammation [110,111]. Signals of physiological and environmental stressors, such as growth factors, cytokines, tumor promoter molecules, and UV radiation, can activate AP-1 (Figure 3B). 

It has been observed that the Fos proteins and members of the Jun family hetero-dimerize; on the other hand, the Jun proteins can form transcriptionally active complexes by both homo- and hetero-dimerizing with the Fos members [112]. When activated, DNA-binding activity and the formation of Jun homo-dimers or Jun/Fos hetero-dimers occur. Interactions with other transcriptional regulators modulate the activity of AP-1, which is further regulated by upstream kinases connecting AP-1 proteins to different signal transduction pathways (Figure 3B). 

The dimeric complex of AP-1, which includes leucine zipper proteins from the JUN, FOS, ATF, and MAF multigene families, is responsible for maintaining fine cell homeostasis [113]. While JUND and JUNB demonstrate tumor-suppressive activity, JUN and FOS frequently display oncogenic characteristics when acting as either tumor suppressors or oncogenes in cancer [113]. In PCa, JUNB, Fos, and JUND are associated with combinations of signaling events, including c-JUN amino-terminal kinase (JNKs), extracellular signal-regulated kinases (ERKs), and other regulatory pathways, like PI3K and the p38 family of kinases, which directly activate the transcription of Jun and Fos and are responsible for activating AP-1 [114]. These signaling events are stimulated by mitogen-activated protein kinases (MAPKs) (Figure 3B) [115,116].

### AP-1 Roles in Prostate Cancer

Human cancers exhibit differential expression of the AP-1 protein family, which may contribute to cancer development. Alterations in the composition of AP-1 complexes are linked to increased proliferation rates, malignant transformation, and tumor aggressiveness [117]. PCa cell lines become more invasive when c-Jun or c-Fos are overexpressed. Also, the phosphorylation of c-Jun is elevated in PCa samples [114], suggesting that the upstream kinases of AP-1 play a key role in PCa initiation and progression [117,118]. 

A stage- and tissue-specific variation in the AP-1 factor expression pattern may be essential to the oncogenesis process. Remarkably, in a mouse model of androgen-independent tumors, it has been proposed that there was a definite elevation of the Fos and Jun proteins. The crosstalk between AP-1 and AR may therefore play a significant role in the initiation and advancement of PCa [114]. 

AP-1 can also trigger proliferation when growth factors and stress signals are activated upstream [119]. Molecular evidence suggests that the network of pro-inflammatory AP-1 and associated chemokines is highly enriched in symptomatic prostate disease [97] and shares similarities with other chronic autoimmune diseases at a molecular level [112]. 

In advanced and metastatic PCa, there is upregulation of c-Jun and c-Fos, which is linked to a poor prognosis and recurrence of the disease [120]. Higher levels of active c-Jun were found to be associated with tumor growth that was resistant to castration in a clinical study [121]. Furthermore, patients with greater active forms of phosphorylated c-Jun expression had shorter relapse-free survival durations than those with lower levels of phosphorylated c-Jun protein expression. Furthermore, activation of Fra-1, Jun-D, and c-Jun is linked to the advancement of PCa [120,122]. Our group also demonstrated that combined aberrant activation of NF-κB and AP-1 in CRPC deregulates IL-6 expression [114,122]. Elevated IL-6 levels in patients with mCRPC predict malignant prostate cancer progression and poor outcomes in patients with localized tumors [123,124] Furthermore, we demonstrated that JunD inhibition results in GADD45α- and γ-dependent induction of cell death and inhibition of tumor growth [110].

An association with 5-α reductase inhibitor (5ARI) treatment and an increased expression of c-FOS have been observed [112,125]. As a result, AP-1 inhibitors are a remarkably effective therapeutic strategy to stop the onset, spread, and invasion of tumors [126]. A key regulator of the development of tumors is the expression of the *Jun/Fos* oncogene protein [127].

Curcumin has a great deal of potential for blocking AP-1 activation by directly interacting with the AP-1 DNA-binding motif [128]. It has also been demonstrated to block JNK activation and to promote cell cycle arrest and apoptosis by regulating the level of c-Jun proteins, a critical component of the AP-1 complex that is primarily activated via phosphorylation by JNK [129]. These analyses have been reported on a cellular level, where curcumin appears to suppress tumor progression through AP-1. These results suggest that curcumin might be a strong AP-1 inhibitor and a potential therapeutic option for PCa treatment [130]. 

## 5. NF-κB, beyond the Inflammation Response

**Figure 4:**
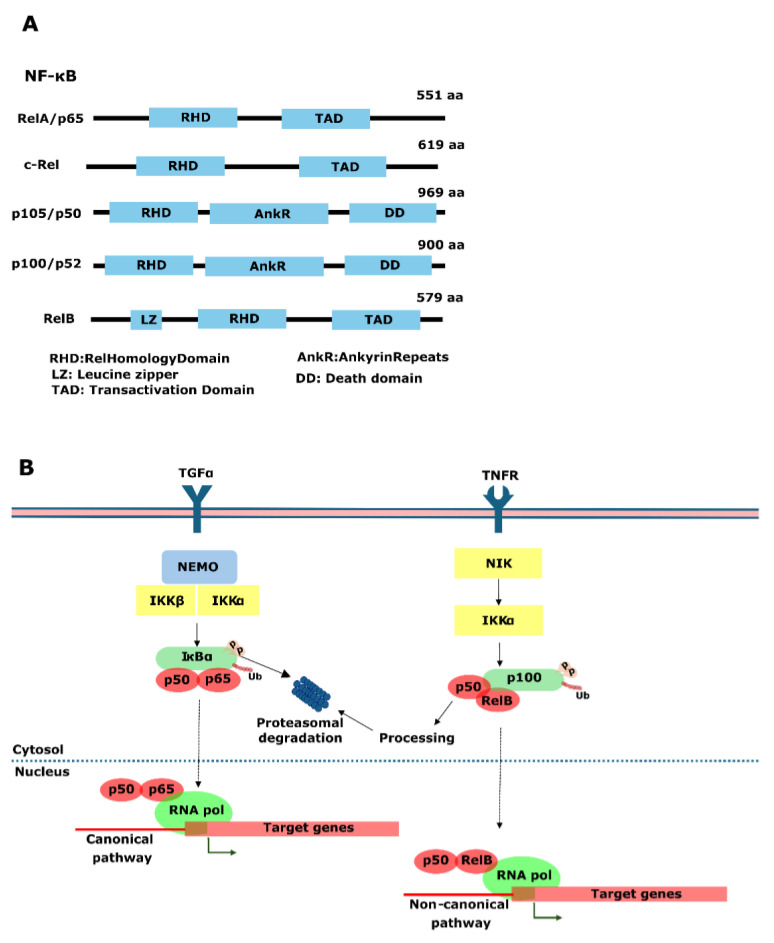
NF-κB and signaling pathways. (**A**) The NF-κB family members, including RelA/p65, C-Rel, P105/p50, P100/p52, and RelB, are characterized by the conserved signature RHD (Rel homology domain). (**B**) Canonical and non-canonical NF-κB signaling pathway. Receptors, such as TLRs, IL-1R, and TNFRs, initiate the canonical pathway. This triggers a cascade leading to the degradation of IκB and the subsequent release of the NF-κB dimer (P50/P65). This dimer is translocated into the nucleus, where it induces the transcription of target genes. On the other hand, the non-canonical pathway is activated by receptors, such as BAFFR, CD40, and RANK. Activation of this pathway triggers a cascade resulting in the release of the p52-RelB dimer, which is then translocated to the nucleus to induce the transcription of target genes.

Eukaryotic transcription factor NF-κB is a family of TFs involved in immune response, inflammation, cell proliferation, differentiation, and cell survival [131]. In humans, the NF-κB superfamily comprises five TFs subdivided in two distinct groups: Rel proteins (REL, RELA, and RELB, also known as c-rel, p65, and p50, respectively) and NFKB proteins (p105 and p100), which can form homo- and hetero-dimeric complexes in almost any combination. The Rel family proteins have C-terminal transactivation domains, whereas the NF-κB proteins have C-terminal ankyrin (ANK) repeat domains (Figure 4A) [132]. Besides these five proteins, the modulation of NF-κB dimer activity is regulated through interaction with a family of inhibitor proteins known as IκBs (Figure 4B) [132].

The NF-κB pathway has two forms of action. The fundamental process of the canonical NF-κB signaling involves a sequence of positive and negative regulatory components. Stimuli that initiate the process lead to the activation of IKK, resulting in the phosphorylation, ubiquitination, and subsequent degradation of IκB proteins. The liberated NF-κB dimers undergo additional activation through diverse posttranslational modifications. These activated dimers then migrate to the nucleus, where they bind to specific DNA sequences and facilitate the transcription of target genes [133]. The noncanonical NF-κB signaling pathway is responsible for activating the p52/RelB NF-κB complex, influencing specific immunological processes. Unlike the canonical pathway, this pathway involves the inducible processing of NF-κB2 precursor protein, p100, rather than the degradation of IκBα. NF-κB-inducing kinase (NIK) and inhibitor of NF-κB kinase α (IKKα) play pivotal roles, leading to phosphorylation-dependent ubiquitination and processing of p100. NIK’s degradation is normally continuous but can be stabilized upon TRAF3 (TNF receptor associated factor 3) degradation in response to signals from certain TNF receptor superfamily members [134]. It is widely recognized that the canonical pathway essential for controlling the inflammatory response, the noncanonical pathway, is regarded as a crucial regulator of the immune response (Figure 4B) [135]. 

The inflammation response is one of the factors that influences the metastasis and therapeutic resistance in PCa. The activation of NF-κB plays a crucial role in regulating the expression of various cytokines and factors associated with cancer development. This activation leads to the expression of interleukins, such as IL-6, for tumor cell survival, as well as angiogenic factors, like VEGF and IL-8. Also, it triggers the production of inflammatory mediators, promoting the recruitment of immune cells. NF-κB activation is observed in multiple cancer types, including PCa, where it is linked to survival, progression, resistance to chemotherapy, and metastasis [136,137,138]. 

Considering the various functions that NF-κB performs in different stages of PCa progression, it emerges as a potent instrument for diagnosis and prognostic and therapeutic interventions. 

### NF-κB in Prostate Cancer

NF-κB p65 has been proven to be a relevant biomarker for several PCa features. Immunohistochemical staining of PC tissues from patients treated through radical prostatectomy targeting NF-κB p65 revealed a strong correlation with the nuclear localization of this molecule and PCa biochemical recurrence (BCR) [139]. Not only a predictor pf BCR, NF-κB p65 is also associated with bone metastasis and PCa-specific death [139]. Also, the constitutive activation of NF-κB p65 has been identified during the progression of CRPC [140].

The NF-kB pathway is the target of several studies that aim to find novel approaches for PCa treatment or optimize the treatments that are already available. Roburic acid, a natural compound derived from oak galls and *Gentiana macrophyla* Pall, demonstrates the ability to inhibit nitric oxide and IL-6 by targeting the NF-kB and MAPK pathway in RAW 264.7 murine macrophages. When combined with docetaxel (the primary chemotherapeutic agent for advanced PCa), roburic acid leads to the suppression of NF-kB and Bcl2 (in the intrinsic apoptosis pathway), along with an upregulation of Bax (apoptosis regulator Bax). This combined effect results in reduced cell growth, sphere formation, migration, and invasion, accompanied by an increase in apoptosis [141]. CRPC is a recurrent condition that usually emerges because of ADT. Dimethylaminopathenolide (DMAPT), a Parthenolide analogue that inhibits NF-κB activity by preventing the degradation of IκB-α and IκB-β, increases the sensitivity of PCa cells to X-rays in vitro and in vivo [142]. In vitro, DMAPT treatment resulted in a recovery of sensibilization to ADT in castration-resistant cell lines through AR and AR-V7 downregulation mediated by NF-κB inhibition [143]. β-elemonic is another compound that also has an impact on castration-resistant cell lines by inducing apoptosis inhibiting JAK2/STAT3/MCL-1 and NF-κB pathways (Figure 4B) [144].

## 6. MYC, a Master Regulator of Transcription

The master TF, MYC (also called c-MYC), is intricately regulated under normal physiological conditions. As with TF, MYC regulates the activity of approximately 15% of all genes [145], playing a pivotal role in maintaining cellular homeostasis. This regulation extends to multiple fundamental biological processes, such as cell cycle progression, apoptosis, metabolism, protein synthesis, cell growth, and cell differentiation, underscoring MYC’s multifaceted influence on cellular dynamics. In cancer, MYC is one of the most frequently deregulated genes. Many of the cancer hallmarks are activated by MYC deregulation, including proliferation, genomic instability, cell survival, self-renewal, metabolism, invasiveness, immune evasion, and angiogenesis [146]. Its deregulation is a potent driver of oncogenesis in other cancer types, including PCa.

**Figure 5:**
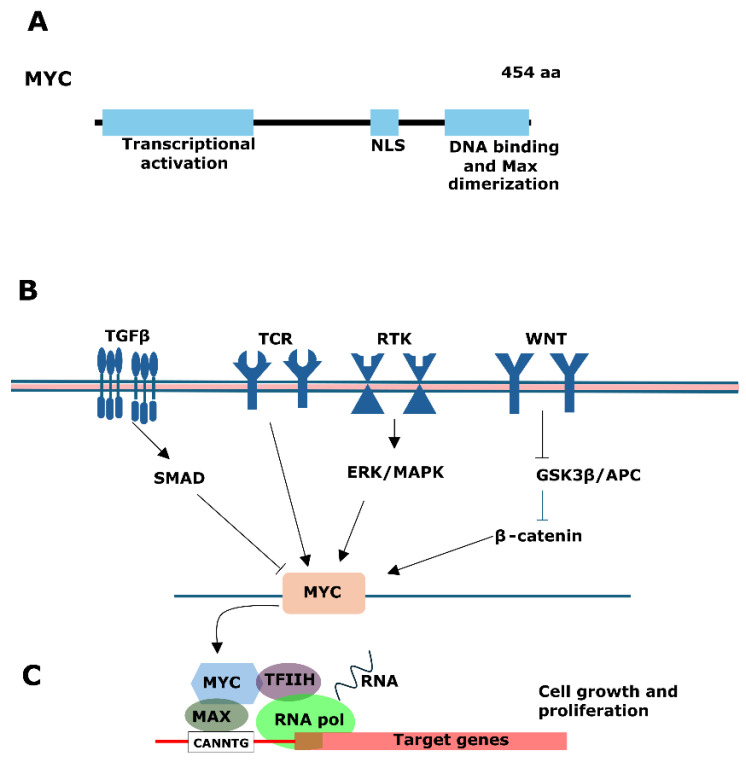
Schematic representation of MYC protein and its signaling pathway. (**A**) The protein structure of MYC includes a transcriptional activation domain, nuclear localization signal (NLS), and DNA binding/MAX dimerization domain. (**B**) Transcriptional activation of MYC expression. MYC transcription is regulated by the receptor signal transduction pathways, including the TGFβ, TCR, RTK, and WNT pathway. (**C**) Regulation of transcription by MYC. MYC binds with Max and binds to the target DNA sequences or E-boxes (with the sequence 5′-CANNTG-3′), thereby controlling the transcription of genes associated with cell growth and proliferation.

To regulate transcription, MYC forms a hetero-dimer with MYC-associated factor X (MAX). The MYC-MAX hetero-dimer can bind to DNA response elements, also known as enhancers, or the E-box region in altering the transcription (Figure 5A) [147]. In addition to its fundamental role in transcription, MYC also contributes to chromatin remodeling and DNA replication (Figure 5B). 

Transcriptional PCa studies indicate that MYC is upregulated in PCa tissue compared to normal prostate tissue or benign prostatic hyperplasia. Furthermore, MYC amplification is associated with a higher Gleason grade and unfavorable prognosis in patients with PCa [148,149]. Interestingly, alterations in MYC are more frequently reported in black men than white men [149,150]. MYC overexpression was identified as an early event in PIN [151]. In mice models, MYC overexpression in normal prostate luminal cell leads to the development of PCa [152]. It has been speculated that these 8q24 genetic variants on the 8q24 chromosome affect MYC expression by altering its regulation or amplification status. Germline variations in this genomic region are reported in about 25% of familial PCa patients [153,154]. The chromosomal region 8q24 is the second most amplified region within CRPC [63]. Together, these results illustrate that MYC is an important driver of PCa (Figure 5B).

### 6.1. Diagnostic Potential of MYC

The idea to use MYC as a diagnostic or prognostic marker lies in the role it plays in PCa development and progression. Studies consistently report that MYC is overexpressed in PCa tissues compared to normal prostate tissues. This aberrant expression is indicative of dysregulation and can serve as a distinguishing factor to distinguish cancer from normal tissue; therefore, it can serve as a diagnostic marker. Elevated tissue levels of MYC have been associated with more aggressive PCa phenotypes. PCa with high MYC expression exhibit increased tumor growth, invasion, and metastasis. Therefore, MYC can potentially be used as a prognostic marker, as assessing MYC tissue levels can contribute to determining the aggressiveness of the disease.

MYC-positive cancer can also be detected through real-time transferrin-based PET (Positron Emission Tomography) scans that utilize Gallium-68 Citrate [155]. In the blood, Gallium-68 Citrate binds to circulation transferrin, and the complex is transported into the cell by the transferrin receptor (TFRC). As MYC is responsible for the transcription of TFRC, cells overexpressing MYC will usually have alleviated levels of TFRC, thereby increasing the uptake of Gallium-68 Citrate [155]. This technique can identify patients who may benefit from MYC-targeted treatment as well as provide a means to measure the activity of MYC transcription in real time with quantitative imaging.

Beyond tissue analysis and imaging, MYC copy-number gain is detectable in cell-free DNA (cfDNA) isolated from blood [149,150] and urine [156], opening the potential of MYC to be used as a circulating biomarker. The detection of MYC levels in bodily fluids, such as blood or urine, may provide a less invasive method for diagnosing and monitoring PCa when compared to biopsy from a single site [156]. In CRPC, MYC copy number gain, detectable in cfDNA analysis, was reported in 20% of patients [149,157] and was associated with a shorter overall survival [157], implying that it may be a key factor to consider in treatment selection towards personalized medicine.

Given that MYC expression correlated with cancer progression, monitoring circulating alterations in MYC over time would offer insight into the advancement of disease. When combined with other markers and clinical assessments, MYC data may enhance the accuracy of the diagnostic and prognostic process.

### 6.2. MYC as a Therapeutic Target

MYC has been termed “undruggable” due to its protein structure that contains regions of intrinsic disorder with the absence of favorable target-binding moieties [158]. Additionally, the protein has a critical function in normal cells [159], which can lead to off-target effects in normal tissue and drug treatment side effects. Complicating matters further, two paralogues (MYCN and MYCL) with overlapping functions but distinct expression patterns are found in vertebrate [160]; for effective inhibition, these need to be targeted as well. Despite these obstacles, several new MYC-directed therapies are in the research and clinical stages of development. The therapies are directed to either reduce MYC levels (by targeting transcription and mRNA translation or by reducing protein stability) or disrupt MYC’s function (by interfering with the protein–DNA or protein–protein interaction, particularly with MAX) [161].

The promoter region of MYC contains a G-quadruplex (G4) motif. Small molecule inhibitors can bind and stabilize the region, thereby reducing transcription of MYC [162]. Several small molecule inhibitors for G4 stabilization are currently under investigation, including Quarfloxin (CX-3543), Pidnarulex (CX-5461), and APTO-253. Although Quarfloxin reached Phase II clinical trials for neuroendocrine carcinoma treatment (NCT00780663) [163], it had poor clinical outcomes. In Phase I clinical trials, both Pidnarulex (NCT04890613 for solid tumor treatment, including PCa) and APTO-253 (NCT02267863) were well-tolerated.

Bromodomain extra terminal (BET) inhibitors are small molecule inhibitors designed to interact with proteins containing bromodomains, such as the TFs MYC (Figure 5A). These inhibitors disrupt the ability of BET proteins to bind to acetylated lysine amino acids on histones, thereby influencing the control of gene transcription. BET inhibitors are being explored as treatments for a range of cancers, particularly those associated with MYC dysregulation. Examples of BET inhibitors include JQ1, I-BET762 (Ibrutinib), OTX015 (MK-8628), RVX-208, and ZEN-3694. A Phase II clinical trial (NCT04471974) is investigating the MYC inhibitor ZEN-3694 in combination with Pembrolizumab and Enzalutamide for the treatment of CRPC. ZEN-3694 treatment led to the downregulation of MYC, GR, and AR signaling that prevent cellular growth. The clinical trial show acceptable tolerance and provide promising preliminary efficacy result [164].

Small molecule inhibitors that disrupt the dimerization between MYC and MAX include the 1-ring heterocyclic compound 10074-G5 and its second-generation congener JY-3-094 and 3JC48-3 [165]. 3JC48-3 is five times more potent than 10074-G5. The inhibition of the c-MYC/MAX hetero-dimer by 3JC48-3 is linked to reduced growth and viability of PCa cells, which is correlated with an increase in kinase protein kinase D1 (PrKD1) expression and kinase activity. Additionally, animal models show favorable tolerability of 3JC48-3 in both patient-derived xenograft and wild-type mouse models, suggesting its potential suitability for advancing into additional preclinical investigations [166,167]. Other small molecule inhibitors that disrupt the MYC-MAX hetero-dimer are MYCi975 [168].

OmoMYC is a synthetic 90 amino acid peptide designed to mimic the structure of the MYC protein [169]. OmoMYC inhibits cancer growth by forming homo-dimers as well as hetero-dimers with MYC and MAX. The OmoMYC homo-dimer and OmoMYC-MAX hetero-dimer bind to DNA but do not activate transcription. Instead, it competes with the binding of the MYC-MAX hetero-dimer to the DNA and subsequently inhibits MYC-mediated transcription of the target genes [170,171]. The binding of OmoMYC to MYC, on the other hand, disrupts the DNA binding domain and thereby prevents transcriptional activation.

A new and intriguing treatment approach in precision medicine is to target oncogenes at the genomic DNA level. Recently, a γ peptide nucleic acid (γPNA)-based genomic DNA-targeted platform was used to silence MYC in cell lines and preclinical mouse models [172]. Interestingly, co-treatment employing histone deacetylase inhibitors and chemotherapeutic drugs demonstrated strong antitumor activity in patient-derived xenografts. This strategy offers an exceptional therapeutic platform aiming to target genomic DNA to inhibit oncogenes. In summary, this approach presents an innovative therapeutic framework aimed at targeting genomic DNA to suppress oncogenes as a means of cancer treatment [172]. MYC exerts such a broad impact on cell metabolism that even elevated fat consumption may exacerbate the characteristics of MYC and intensify its transcriptional activities. The MYC signature triggered by fat intake could be valuable for identifying patients at greater risk of developing more aggressive and fatal PCs. Patients exhibiting this MYC signature may benefit from epigenetic treatments directed at MYC or dietary interventions aimed at the metabolic requirements controlled by MYC [173].

## 7. Concluding Remarks

As a global regulator of protein expression, imbalance of TF expression is linked to the development of diseases, such as cancer. In PCa, this imbalance can result in more aggressive forms of disease, and it is linked to the development of drug resistance disease, considerably worsening patients’ prognostics. Because TFs permeate cell metabolism in a very intricate manner, they may lead to either increased carcinogenesis or inhibition of tumor progression, and strategies for their suppression or overexpression are being historically employed. As discussed in this review, the mechanisms of action of different TFs are peculiar. They can act alone in triggering different pathways, but they can also control the expression of other TFs (as observed for AR/ERG and ERG/AP-1, for example). The diversity in TFs’ signaling pathways and the difficulty in understanding their precise role in PCa have meant that they have been considered “undruggable” for a long time. The complex interaction between TF and DNA represents a significant hurdle, as designing molecules to selectively inhibit these interactions without disrupting essential cellular processes is challenging. In addition, target specificity is complicated because TF regulates multiple downstream genes, which can lead to undesirable consequences and off-target effects, and its intracellular localization complicates drug delivery. Also, lack of well-defined binding pockets and the fact that TFs have large, flexible structures unsuitable for small molecule binding present a significant obstacle for drug development. Moreover, cellular transcriptional plasticity and redundancy can limit the efficiency of therapeutic efficacy by compensating for the loss of targeted TFs or through overlapping functions within TF families.

Different approaches can be employed to inhibit TFs, such as, for example, direct targeting aiming to inhibit DNA-binding activity or disrupting essential protein–protein interactions in TFs’ function. However, achieving specificity and selectivity for the target TF while avoiding off-target effects is still a challenge. In this sense, indirect inhibition via modulation of upstream pathways or post-translational modifications that regulate TF activity may be an alternative. Furthermore, genetic inhibition via RNA interference (RNAi) and CRISPR-based approaches for selectively silencing TF expression can lead to efficient outcomes, although challenges, such as delivery and off-target effects, need to be addressed for clinical translation.

More than treatment targets, another feature that has been explored is their use for diagnostics and prognostic purposes. Because the seminal report of Tomlins [52] correlated higher frequency of translocations with the aggressive phenotype of PCa, identifying the expression/suppression of TFs in different stages of PCa development or even as a predictor of response to treatment may have a huge impact on decision making for therapy. In this sense, it is important to highlight that the differences in the genetic makeup of different populations may influence the level of expression or the frequency of translocations of otherwise relevant PCa TFs. These studies are essential for the development of population-specific diagnostic/prognostic and therapeutic approaches.

The studies of TFs and their TFs interactions are still being use for the development of prognostic and therapeutic approaches. Prognostic data from genomic classifiers or molecular biomarkers should be incorporated into standard clinical parameters if doing so would affect short- or long-term clinical management to close these gaps and enhance risk stratification and treatment management of PCa patients. Interestingly, clinical trials evaluating TFs for diagnostic, prognostic, and therapeutic purposes are common and promising, as observed regarding the elevated number of clinical trials in the last five years (see Table 1 for a summary of active trials in the mentioned period).

Furthermore, we observed a huge tendency in studies focusing on the use of TFs’ target molecules as adjuvant therapy for patients receiving ADT. This approach can lead to resensitization of CRPC to ADT, therefore regaining its efficacy, and it is a good example of multi-target approaches for PCa treatment. TFs in PCa have being extensively studied, but their role as drug targets or even as diagnostic or prognostic players is still not fully elucidated, which makes it a hot topic. The diversity in interactions of these molecules is still a challenge for the development of specific drugs and multitarget approaches. Although challenging, the clinical trials in the last five years suggest that TFs are promising therapeutic targets and reliable markers for diagnosis and prognosis.

## Figures and Tables

**Table 1:** Phase III and IV in clinical trials and observational studies related to transcription factors (last 5 years).

Study ID	TF	Treatment	Phase	Status	Application of TF in the Study
NCT04689828	AR	177Lu-PSMA-617/68Ga-PSMA-11/ARDT	Phase III	Active/not recruiting	Comparison of 177Lu-PSMA-617 vs. a change in androgen-receptor-directed therapy in the Treatment of taxane-naïve men with progressive mCRPC
NCT04720157	AR	177Lu-PSMA-617/68Ga-PSMA-11/ARDT/ADT	Phase III	Active/not recruiting	In the study, the SoC is defined as a combination of ARDT + ADT
NCT05352178	AR	ADT/AR targeted therapy/radiotherapy and/or surgery	Phase III	Recruiting	Adjustment of ADT treatment cycles in PMFS and/or mCRPCFS patients
NCT05191680	AR	Apalutamide/placebo	Phase III	Recruiting	Use of short-term androgen deprivation therapy in the form of apalutamide (Erleada) in men in active surveillance for prostate cancer
NCT03601143	AR	Observational study	N/A	Recruiting	Determines the optimal method to determine AR-V7 status. Also investigates AR-V7-independent mechanisms of resistance and their predictive value for proper treatment
NCT04647526	AR	[Lu-177]-PNT2002/Abiraterone/Enzalutamide	Phase III	Active/not recruiting	Evaluates the efficacy and safety of [Lu-177]-PNT2002 in patients with metastatic mCRPC who have progressed following treatment with ARAT
NCT06136650	AR	MK-5684/dexamethasone/fludrocortisone acetate/hydrocortisone/Abiraterone acetate/prednisone acetate/Enzalutamide	Phase III	Recruiting	Study of MK-5684 versus alternative NHA in mCRPC post one NHA (MK-5684-004)
NCT03665922	MYC/AR	BroccoMax/placebo	N/A	Active/not recruiting	MYC and AR as a marker to evaluate prostate adenocarcinoma
NCT04601441	MYC/ERG	Apalutamide	Phase IV	Recruiting	Evaluation of genomic alterations of 73 PCa driver genes between pre- and posttreatment
NCT02573636	ERG	Observational study	N/A	Recruiting	Evaluates the predictive value of *TMPRSS2-ERG* gene fusion in patients with high-risk Pca treated with first-line LHRHa after biochemical failure
NCT05612880	AR	Observational study	N/A	Recruiting	Observational study for the determination of longitudinal effects of androgen receptor signaling inhibitors (ARSI) in men with advanced PCa
NCT04484818	AR	Darolutamide/Goserelin acetate/Leuprolide acetate/placebo/Triptorelin	Phase III	Active/not recruiting	Androgen receptor activity as a prognostic signature
NCT05819606	AR	Observational study/immunohistochemistry analysis	N/A	Not yet recruiting	AR expression as a tool to evaluate the progress of the treatment
NCT04769817	AR/ERG	Observational study	N/A	Recruiting	AR as a prognostic biomarker
NCT03903835	AR/ERG	Enzalutamidde/Abiraterone/Carboplatin/Cabazitaxel/Docetaxel/Radium Chloride Ra223/Niraparib + Abiraterone + Prednisone/Capivasertib+Docetaxel/Apalutamide/Darolutamide	Phase III	Recruiting	Androgen receptor as a biomarker signature
NCT03784924	ERG	MRI (diagnostics)/Observational study	N/A	Recruiting	ERG used in the study as a diagnostic tool

All of the studies cited above are available on the clinicaltrials.gov website by searching with the terms “prostate cancer”, “NF-κB”, “PTEN”, “MYC”, “AP-1”, “androgen receptor”, “SPDEF”, and “ERG” combined with the selection of the following filters: “Looking for participants: not yet recruiting/recruiting”, “No longer looking for participants: active, not recruiting”, “Sex: male”, “Studies from 1 January 2019 to 1 January 2024”, and “Age range: adult/older adult”. Only advanced phase (III and IV) and observational studies were selected.

## Data Availability

Not applicable.
